# Tris DBA ameliorates IgA nephropathy by blunting the activating signal of NLRP3 inflammasome through SIRT1‐ and SIRT3‐mediated autophagy induction

**DOI:** 10.1111/jcmm.15663

**Published:** 2020-11-01

**Authors:** Chung‐Yao Wu, Kuo‐Feng Hua, Shin‐Ruen Yang, Yi‐Shan Tsai, Shun‐Min Yang, Chih‐Yu Hsieh, Chia‐Chao Wu, Jia‐Feng Chang, Jack L. Arbiser, Chiz‐Tzung Chang, Ann Chen, Shuk‐Man Ka

**Affiliations:** ^1^ Graduate Institute of Life Sciences National Defense Medical Center Taipei Taiwan; ^2^ Department of Biotechnology and Animal Science National Ilan University Ilan Taiwan; ^3^ Department of Pathology Tri‐Service General Hospital National Defense Medical Center Taipei Taiwan; ^4^ Department of Internal Medicine En Chu Kong Hospital New Taipei City Taiwan; ^5^ Renal Care Joint Foundation New Taipei City Taiwan; ^6^ Division of Nephrology Department of Internal Medicine Tri‐Service General Hospital National Defense Medical Center Taipei Taiwan; ^7^ Department of Dermatology Emory School of Medicine and Winship Cancer Institute Atlanta GA USA; ^8^ Atlanta Veterans Administration Medical Center Decatur GA USA; ^9^ Division of Nephrology Department of Internal Medicine China Medical University Hospital Taichung Taiwan; ^10^ Graduate Institute of Aerospace and Undersea Medicine Department of Medicine National Defense Medical Center Taipei Taiwan

**Keywords:** autophagy, IgA nephropathy, NLRP3 inflammasome, SIRT1, SIRT3, Tris (Dibenzylideneacetone) dipalladium

## Abstract

Tris (dibenzylideneacetone) dipalladium (Tris DBA), a small‐molecule palladium complex, can inhibit cell growth and proliferation in pancreatic cancer, lymphocytic leukaemia and multiple myeloma. Given that this compound is particularly active against B‐cell malignancies, we have been suggested that it can alleviate immune complexes (ICs)–mediated conditions, especially IgA nephropathy (IgAN). The therapeutic effects of Tris DBA on glomerular cell proliferation and renal inflammation and mechanism of action were examined in a mouse model of IgAN. Treatment of IgAN mice with Tris DBA resulted in markedly improved renal function, albuminuria and renal pathology, including glomerular cell proliferation, neutrophil infiltration, sclerosis and periglomerular inflammation in the renal interstitium, together with (*Clin J Am Soc Nephrol*. 2011, *6*, 1301‐1307) reduced mitochondrial ROS generation; (*Am J Physiol‐Renal Physiol*. 2011. *301*, F1218‐F1230) differentially regulated autophagy and NLRP3 inflammasome; (*Clin J Am Soc Nephrol*. 2012, *7*, 427‐436) inhibited phosphorylation of JNK, ERK and p38 MAPK signalling pathways, and priming signal of the NLRP3 inflammasome; and (*Free Radic Biol Med*. 2013, *61*, 285‐297) blunted NLRP3 inflammasome activation through SIRT1‐ and SIRT3‐mediated autophagy induction, in renal tissues or cultured macrophages. In conclusion, Tris DBA effectively ameliorated the mouse IgAN model and targeted signalling pathways downstream of ICs‐mediated interaction, which is a novel immunomodulatory strategy. Further development of Tris DBA as a therapeutic candidate for IgAN is warranted.

## 1 INTRODUCTION

IgA nephropathy (IgAN) is the most prevalent primary glomerulonephritis worldwide, featuring mesangial cells and mononuclear leucocyte infiltration in the glomerulus and renal interstitial tissues.^1^, ^2^, ^3^ The renal disorder has been shown to progress to uraemia in 20%‐40% of the patients.^4^, ^5^ Clinically, current treatment for IgAN and its progression of disease is still insufficient, because the benefits of these non‐disease targeted drugs are modest. Most current therapies target production of antibodies non‐selectively, rather than the effect of immune complexes (ICs) directly. Therefore, novel therapeutics are urgently needed for the treatment of this renal disorder. Recently, we demonstrated that overproduction of reactive oxygen species (ROS) and mononuclear leucocyte infiltration into the kidney is involved in the development and progression of IgAN.^6^, ^7^, ^8^ Furthermore, we^9^, ^10^ and others^11^ have shown that NLRP3 inflammasome are implicated in the development and deterioration of IgAN. On the other hand, we showed that activation of MAPK signalling pathways in mesangial cells and macrophages and T cells infiltrated in the glomerulus may be involved in the pathogenesis of IgAN.^12^ Activation of NLRP3 inflammasome results in the production of IL‐1β and IL‐18, involving a priming signal from pathogen‐associated molecular patterns and an activation signal.^13^, ^14^, ^15^ In this regard, ICs can initiate the activation of the NLRP3 inflammasome in macrophages in IgAN.^6^, ^7^, ^8^ Recently, we have shown overproduction of IL‐1β or IL‐18 in mouse IgAN models, pointing to NLRP3 inflammasome and related signalling pathways as a major mechanism underlying the progression and deterioration of IgAN.^6^, ^7^, ^8^

Mammalian SIRT1 deacetylates various target proteins relevant to apoptosis, cell cycle, circadian rhythms, mitochondrial function and metabolism. Growing evidence suggests the beneficial effects of sirtuin (SIRT) 1 and 3, which are NAD‐dependent deacetylases. SIRT1 and SIRT3 are able to inhibit IL‐1β expression and the production of reactive oxygen species (ROS),^16^, ^17^ and therefore, it is implicated in autophagy induction.^18^ Activation of SIRT1 can inhibit NLRP3 inflammasome activation and subsequent IL‐β secretion, and SIRT1 knockdown can enhance the activation of NLRP3 inflammasome in cultured endothelial cells.^19^, ^20^ In addition, SIRT3 is renoprotective by inhibiting mitochondrial ROS production and NLRP3 inflammasome activation.^21^ Activation of SIRT1 can inhibit NLRP3 inflammasome activation and subsequent IL‐β secretion, and SIRT1 knockdown can enhance the activation of NLRP3 inflammasome in cultured endothelial cells.^19^, ^20^ In addition, SIRT3 is renoprotective by inhibiting mitochondrial ROS production and NLRP3 inflammasome activation.^21^ Autophagy is being regulated precisely and plays a vital role in maintaining cell homeostasis. It is a typical protective, pro‐survival response at the initiation of damages.^22^ Autophagy is involved in the inhibition on IL‐1β secretion^23^, ^24^ and degrading pro‐IL‐1β expression in macrophages.^25^, ^26^ On the other hand, SIRT1 and SIRT3 are implicated in mTOR nutrient‐sensing pathways and regulating autophagy machinery depending on ATG5 and LC3B in autophagy induction in macrophages and liver cells.^27^ In addition, autophagy inhibits NLRP3 inflammasome and innate immune response and inflammation,^28^, ^29^ from which evidence supports that autophagy plays a negative regulator in the activation of NLRP3 inflammasome for the restoration of tissue homeostasis after damage in immune‐mediated diseases, including IgAN.^10^, ^11^, ^30^

Tris (Dibenzylideneacetone) dipalladium (Tris DBA) is a small‐molecule palladium complex, and it has been shown to suppress cell growth and proliferation of pancreatic cancer, lymphocytic leukaemia and multiple myeloma.^31^, ^32^ Tris DBA can reduce Src/NMT‐1 complex in melanoma cells and inhibit its downstream signalling, such as MAPK.^33^ However, the effects of Tris DBA on inflammatory disease have yet to be determined. Given that IgAN is mediated in large part by the proliferation and activation of B cells, and Tris DBA has been shown to be effective against B‐cell malignancy, we evaluated it against IgAN. This is the first study to evaluate the efficacy of this agent against an ICs‐mediated chronic kidney disease.

In the present study, we showed the therapeutic effect of Tris DBA on IgAN in a mouse model and the mechanism of action of the compound, involving inhibition of a MAPK‐mediated priming signal of NLRP3 inflammasome and enhancement of SIRT1 and SIRT3‐mediated induction of autophagy and subsequent NLRP3 inflammasome suppression. This compound targets ICs‐macrophage interactions as a novel immunomodulatory event. These results suggest that the pure compound be a drug candidate for treating IgAN.

## 2 MATERIALS AND METHODS

### 2.1 Tris DBA and optimal dose selection

Tris DBA palladium was purchased from Sigma‐Aldrich and dissolved in KATIMIN (China Chemical & Pharmaceutical Co.) for in vivo studies or in DMSO for in vitro studies.

### 2.2 In vivo experiments

#### 2.2.1 IgAN mouse model and experimental protocol

Reference^1^ two normal control groups were included in this study as follows: 8‐week‐old C57BL/6 mice were injected with saline and served as saline control, while those injected with vehicle (KATIMIN) were served as vehicle control^2^; groups for IgAN and IgAN with treatment were as follows: IgAN was induced by consecutive 28 daily injections of purified IgA anti‐phosphorylcholine antibodies and pneumococcal C‐polysaccharide antigen (PnC) as described previously.^8^, ^9^, ^10^ On day 7 after disease induction, the mice were divided into 3 groups and administered daily either Tris DBA (30 mg/kg bodyweight) or vehicle (KATIMIN) or saline via an intraperitoneal route throughout the study. The animals were killed at days 28 and all animals survived during the entire course of the study, and there was no difference in bodyweight detected among Tris DBA and untreated control groups during the 4‐week dosing period. We used 7 mice per group throughout the study.

All animal experiments were performed with the approval of the Institutional Animal Care and Use Committee of the National Defense Medical Center, Taipei, Taiwan, and in compliance with the NIH Guidelines for the Care and Use of Laboratory Animals.

#### 2.2.2 Urine albumin and renal function measurement

Serum samples were collected to measure the levels of BUN and Cr Urine samples were collected weekly, and albuminuria was determined by the ratio of urine albumin to urine Cr, as described previously.^9^, ^10^, ^34^

#### 2.2.3 Renal pathology and IHC

For histopathological assessment, renal cortical tissue samples were fixed in 10% buffered formalin and embedded in paraffin for haematoxylin and eosin staining. Fifty glomeruli were examined in at least two renal tissue fields of view per slide under a light microscope at a magnification of 400×. Cell proliferation, glomerular sclerosis, neutrophil infiltration and periglomerular mononuclear leucocyte infiltration were recorded as described previously.^4^ For IHC, F4/80 (Serotec, CA, USA) and CD3 (Dako, Glostrup, Denmark Dako) were used in formalin‐fixed and paraffin‐embedded renal sections, followed by incubation with biotinylated secondary antibodies and avidin‐biotin‐peroxidase complexes (both from Dako) as described previously.^4^, ^9^, ^10^

An image analysis software (Pax‐it; Paxcam) (https://www.paxit.com) was used to detect and sort objects or areas, and to quantify the number of positive cells for CD3^+^ and F4/80^+^ expression in twenty randomly selected fields of the glomeruli and periglomerular interstitial compartments, respectively, at a magnification of 400× by light microscopy.^35^

#### 2.2.4 Western blot analysis

Cytoplasmic protein was extracted from renal tissues and cultured cells and immunoblotting using antibodies against mouse NLRP3, caspase‐1 (GeneTex), IL‐1β (R&D Systems, Minneapolis, MN, USA), IL‐18 (Santa cruz), NADPH p47^phox^ (Santa Cruz), NQO1 (Abcam, Bristol, UK), ATG5 (MBL, Japan), LC3B (GeneTex), p62 (Santa cruz), SIRT1 (Cell Signaling), SIRT3 (Cell Signaling), p‐ERK (Cell Signaling), p‐JNK (Cell Signaling), p‐p38 (Cell Signaling) and horseradish peroxidase (HRP)‐conjugated IgG antibodies (Santa cruz) as described previously.^9^, ^10^ β‐actin (Santa Cruz) was used as an internal control for cytosolic protein.

#### 2.2.5 Real‐time PCR analysis

RNA was extracted using REzol (Protech Technology, Taipei, Taiwan), and real‐time PCRs were performed using SYBR Green RT‐PCR Reagents Kit (Applied Biosystems) prepared as described previously.^9^ The specific primer pairs used for real‐time PCR analysis were as follows: mouse NLRP3 forward: 5′ CTGTG TGTGG GACTG AAGCA C‐3′; mouse NLRP3 reverse: 5′‐ GCAGC CCTGC TGTTT CAGCA C‐3′; mouse IL‐1β forward: 5′‐CCAGGATGAGGACATGAGCACC‐3′; mouse IL‐1β reverse: 5′‐TTCTCTGCAGACTCAAACTCCAC‐3′; and mouse Caspase‐1 forward: 5′‐ ACTGTACAACCGGAGTAATACGG‐3′; mouse Caspase‐1 reverse: 5′‐ CACGGA AGGCCATGCCAGTGA‐3′; mouse GAPDH forward: 5′‐TCCGCCCCTTCTGCCGATG‐3′; mouse GAPDH reverse: 5′‐CACGGAAGGCCATGCCAGTGA‐3′.

#### 2.2.6 ROS detection

At ROS levels in renal tissues, the samples were incubated at room temperature with Krebs‐Hepes buffer containing 1.25 mmol/L lucigenin (Sigma) as substrate immediately after the mice were killed, and luminescence counts were determined in duplicate at multilabel microplate reader (Hidex), as described previously.^36^ ROS activity was expressed as relative luminescence units (RLU) per 15 minutes per milligram of the dry weight of the renal tissue (ie RLU/15min/mg) or as RLU/15 min/mL. For the 10 μm of frozen section slides, in situ superoxide anion production was determined in the mouse kidneys by DHE labelling dye (30 μmol/L). Renal tissues were incubated the dye for five min in a dark chamber on an orbital shaker at room temperature, followed by observing under a fluorescence microscope system (Olympus). For the quantified by counting the percentage of the total nuclei that were positive per kidney cross section as described previously.^9^, ^10^ Mitochondrial ROS levels in BMDMs, isolated from C57BL/6, measured by MitoSOX‐based flow cytometry (Thermo) after Tris DBA treatment, priming with IgA‐ICs for 5.5 hours and stimulation with ATP. The data are expressed as the means ± SEM for three separate experiments.

#### 2.2.7 GPx and NF‐κB activity assay

GPx activity in the renal tissue was measured using a commercial glutathione peroxidase assay kit (Cayman, Michigan, USA) according to the manufacturer's instructions as described previously,^4^ and nuclear NF‐κB p65 activation was quantified using an ELISA‐based TransAM NF‐κB kit (Active Motif) according to manufacturer's instructions.

### 2.3 In vitro experiments

#### 2.3.1 Cell models

Murine macrophage cell line J774A.1 was purchased from the American Type Culture Collection (Rockville, MD). The cells were cultured in RPMI 1640 medium supplemented with 10% heat‐inactivated foetal calf serum and 2 mL l‐glutamine (Life Technologies) at 37°C in a 5% CO_2_ incubator. Briefly, 1 × 10^6^ cells/mL was incubated for 30 minutes with or without Tris DBA before them primed with IgA immune complexes (IgA‐ICs) prepared with the purified IgA anti‐phosphorylcholine (5 ng/mL) and PnC (100 ng/mL) was used to stimulate of macrophages for 5.5‐hour serves as NLRP3 inflammasome priming signal including NLRP3 and pro‐IL‐1β activation), and then for 30 minutes with 5 mmol/L ATP serves as NLRP3 inflammasome activation signal including IL‐1β production and caspase‐1 activation. Bone marrow–derived macrophages (BMDMs) were used for ex vivo experiments to measure their levels of mitochondrial ROS production as reported previously.^29^, ^37^

#### 2.3.2 Stable expression of SIRT3 shRNA

Lentivirus transduction particles carrying shSIRT3 (National RNAi Core Facility, Academia Sinica, Taipei, Taiwan) in J774A.1 macrophages were constructed, according to manufacturer's instructions. The cells were then infected with lentivirus‐bearing specific shRNAs of SIRT3 and incubated with puromycin (Invitrogen) to select stably infected cells for further experiments.

#### 2.3.3 Detection of autophagy by confocal laser scanning microscope

J774A.1 macrophages were incubated with or without 3‐MA (5 mmol/L) for 30 minutes, and added with or without Tris DBA (1 µmol/L) for 0.5 hour The cells were then incubated with IgA‐ICs for 3 hours MDC or AO (SIGMA) were added onto slides separately for 10 minutes or 30 minutes followed by washed in PBS. The slides were then fixed in 4% paraformaldehyde for 30 minutes followed by washed in PBS. DAPI for nuclei staining. Image analysis was performed using the confocal microscope (FV1000‐IX81, Olympus).

### 2.4 Data analysis

The data are presented as the mean ± standard error of the mean (SEM), and comparisons between two groups were performed using Student's *t* test. The data from in vitro and ex vivo experiments were analysed using one‐way ANOVA and subsequent Scheffe's test. A *P* value < .05 was considered statistically significant for each of the experiments.

## 3 RESULTS

### 3.1 Renal function, proteinuria and renal pathology

**Figure 1:**
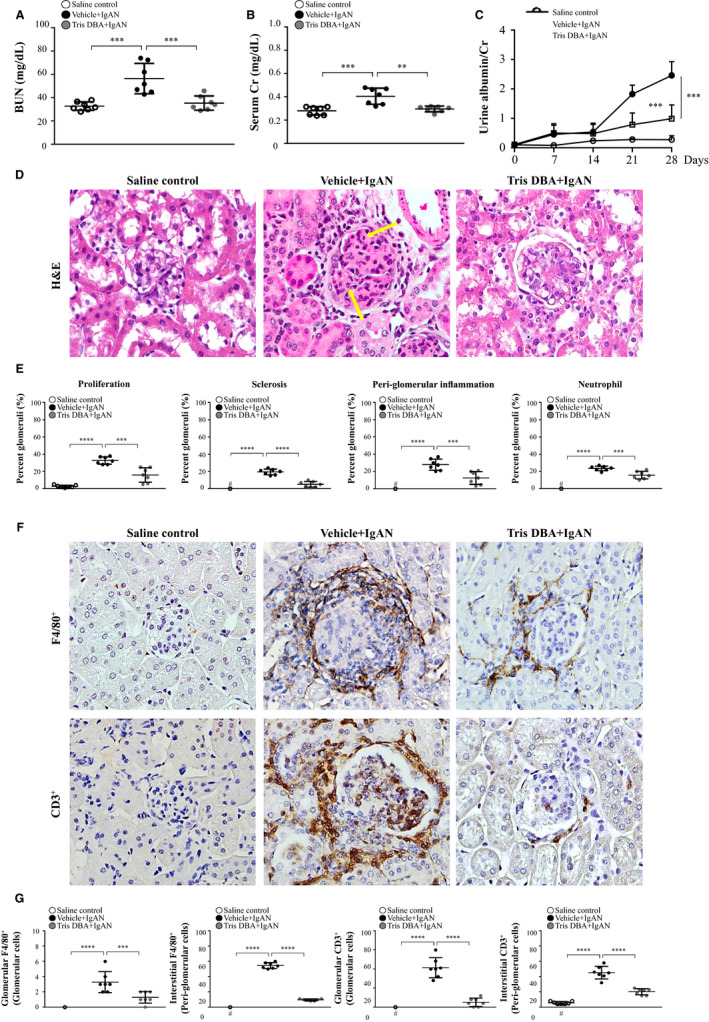
Renal function, renal pathology and T cells and macrophages infiltration. Levels in the serum (A) BUN and (B) Cr; (C) Albuminuria. D, H&E staining. E, Renal lesion scores. F, CD3^+^ T cells and F4/80^+^ macrophages; G, Scoring of CD3^+^ T cells and F4/80^+^ macrophages in IHC staining; Original magnification, 400×. Arrow indicates neutrophils. The data are expressed as the mean ± SEM results in 7 mice per group. #Not detectable, **P < .01, ***P < .005, ****P < .001

To validate the potential therapeutic effects of Tris DBA on IgAN mice, first, we performed clinical assessment and renal pathology. To assess renal function, serum levels of BUN and Cr were determined. As shown in Figure 1A,B, significantly improved renal function was seen in IgAN mice treated with Tris DBA (Tris DBA + IgAN mice) compared to that of IgAN mice treated with vehicle only (Vehicle + IgAN mice). In parallel, although the levels of albuminuria were greatly increased in Vehicle + IgAN mice, compared with those of normal control mice treated with saline (saline control mice) on days 21 and 28, (Figure 1C), this effect was markedly inhibited in Tris DBA + IgAN mice. Additionally, although Vehicle + IgAN mice developed marked glomerular proliferation, mainly mesangial cells, and focal, but intense, glomerular sclerosis, as well as scattered neutrophil infiltration in the glomerulus, along with periglomerular mononuclear leucocytes, and the level of these pathological lesions in the kidney was markedly reduced in the Tris DBA + IgAN mice (Figure 1D,E). By immunohistochemistry (IHC), the phenotype and distribution of mononuclear leucocytes that infiltrated in the kidney were determined. The results showed that marked infiltration of CD3^+^ T cells and F4/80^+^ monocytes/macrophages (Figure 1F‐G) in Vehicle + IgAN mice compared to saline control mice. However, Tris DBA + IgAN mice exhibited significantly reduced the infiltration of these mononuclear leucocytes in renal tissues compared to the Vehicle + IgAN mice.

### 3.2 NLRP3 inflammasome activation in renal tissues

**Figure 2:**
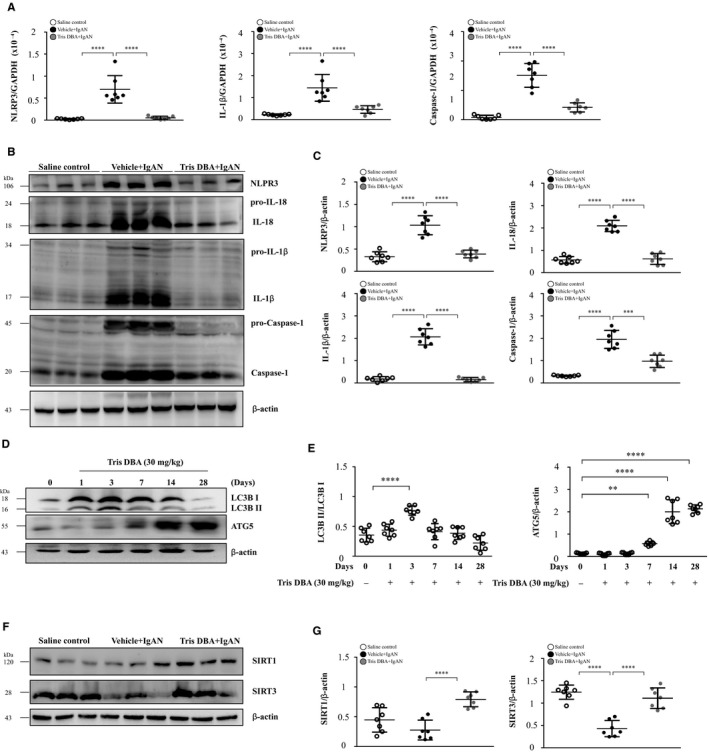
Tris DBA enhanced autophagy induction and inhibited NLRP3 inflammasome activation in IgAN mice. The expression levels of (A) NLRP3, IL‐1β and caspase‐1 in real‐time PCR analysis and (B)NLRP3, IL‐18, IL‐1β and caspase‐1 in Western blot analysis and (C) Semiquantitative analysis. D, Renal LC3B I/II and ATG5 levels after administration of 30 mg/kg Tris DBA to untreated WT mice assessed by Western blot analysis and (E) semiquantitative analysis. F, Renal SIRT1 and SIRT3 levels in Western blot analysis and (G) semiquantitative analysis. Bars show the mean ± SEM results in 7 mice per group. **P < .01, ***P < .005, ****P < .001

In the past, we showed that NLRP3 inflammasome is activated in the mouse IgAN model.^4^, ^9^, ^10^ We examined whether Tris DBA could inhibit renal NLRP3 inflammasome activation in Tris DBA + IgAN mice. The results show that significantly reduced renal mRNA levels of NLRP3, IL‐1β and caspase‐1 were observed in Tris DBA + IgAN mice compared with Vehicle + IgAN mice, although the latter exhibited greatly increased expression levels of all the mRNAs in relevance to saline control mice (Figure 2A, Table 1). Similarly, the Tris DBA + IgAN mice showed significantly reduced renal protein levels of all these proteins compared with Vehicle + IgAN mice, although the latter exhibited greatly increased expression levels of all the proteins in relevance to saline control mice (Figure 2B,C, Table 1). Autophagy can regulate the activation of NLRP3 inflammasome in renal injury.^23^, ^24^, ^25^, ^26^ As shown in Figure 2D,E, renal levels of LC3B I/II protein were markedly elevated with time until day 3 after the treatment with Tris DBA, and then declined on days 7, 14 and 28. Next, we evaluated whether Tris DBA could modulate the induction of autophagy to inhibit activation of the NLRP3 inflammasome in renal tissues from IgAN model. The results show that renal LC3B‐I/II and Atg5 protein levels were significantly higher in Vehicle + IgAN than in saline control, whereas significantly decreased levels of these proteins were observed in Tris DBA + IgAN mice compared with Vehicle + IgAN mice (Figure S2). It is likely that Tris DBA treatment exerted autophagy induction activity under normal conditions, which helped to prevent disease development or progression. However, Tris DBA treatment ameliorated the effect in IgAN mice, presumably followed by a decreased autophagic response due to the improved renal conditions caused by treatment with the compound. Although the exact mechanism is worth further investigation under the disease condition, the emergence of complicated interactions among autophagy induction remains uncertain, and NLRP3 inflammasome activation and compensatory reactions might have occurred in IgAN mice. As shown in Figure 2F,G, the results show that treatment with Tris DBA significantly increased SIRT1 and SIRT3 levels in Tris DBA + IgAN mice, compared with Vehicle + IgAN mice.

**Table 1:** Effects of Tris DBA on NLRP3 inflammasome activation and production of ROS and antioxidants

Groups	Items								
	NLRP3 inflammasome activation				ROS production and antioxidant activation				
	NLRP3	IL‐1β	Caspase‐1	IL‐18	p47 ^phox^	NQO1	GPx	ROS	DHE
Saline control	0.32 ± 0.042	0.18 ± 0.10	0.32 ± 0.028	0.56 ± 0.15	0.65 ± 0.10	2.23 ± 0.38	201.7 ± 14.41	131.2 ± 33.67	21.85 ± 5.96
Vehicle control	‐	‐	‐	‐	0.63 ± 0.05	1.93 ± 0.51	202.1 ± 23.84	116.9 ± 31.90	19.58 ± 10.01
Saline + IgAN	‐	‐	‐	‐	1.074 ± 0.20^§^, ^¶^	0.50 ± 0.14^§^, ^¶^	56.40 ± 16.75^§^, ^¶^	345.5 ± 62.61^§^, ^¶^	113.6 ± 15.29^§^, ^¶^
Vehicle + IgAN	1.03 ± 0.08^†^	2.06 ± 0.36^†^	1.95 ± 0.40^†^	2.097 ± 0.25^†^	1.11 ± 0.11^†^, ^‡^	0.51 ± 0.11^†^, ^‡^	52.11 ± 13.40^†^, ^‡^	348.4 ± 70.80^†^, ^‡^	117.9 ± 9.82^†^, ^‡^
Tris DBA + IgAN	0.3900 ± 0.03*	0.15 ± 0.08*	0.97 ± 0.27*	0.62 ± 0.24*	0.21 ± 0.08*, #	2.21 ± 0.31*, #	208.6 ± 18.39*, #	205.3 ± 50.77*, #	63.34 ± 19.90*, #

Note

The data indicated mean ± SEM results in 7 mice per group; Western blot analysis in NLRP3, IL‐1β, Caspase‐1, IL‐18, p47 ^phox^ and NQO1; GPx detected with glutathione peroxidase activity; ROS detected with relative luminescence units (RLU); immunofluorescence staining analysis in DHE staining.

* Tris DBA + IgAN vs Vehicle + IgAN.

^#^ Tris DBA + IgAN vs Saline + IgAN.

^†^ Vehicle + IgAN vs Saline control.

^‡^ Vehicle + IgAN vs Vehicle control.

^§^ Saline IgAN vs Saline control.

^¶^ Saline IgAN vs Vehicle control.

### 3.3 ROS production in renal tissues and bone marrow–derived macrophages (BMDMs)

**Figure 3:**
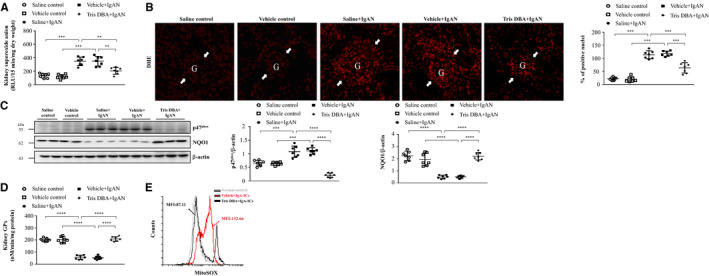
Tris DBA reduced oxidative stress and enhanced ROS activity in IgAN mice. A, Superoxide anion in renal tissues; B, DHE staining on renal tissues and the quantified results of DHE staining. Original magnification, 400X. Arrow indicates glomerular area; C, the protein level of NADPH^p47^ and NQO1 in Western blot analysis in renal tissue and semiquantitative analysis; D, renal GPx level in ELISA. Bars show the mean ± SEM results in 7 mice per group. E, Mitochondrial ROS levels in BMDMs measured by MitoSOX after Tris DBA treatment, priming with IgA‐ICs for 5.5 h and stimulation with ATP. The data are expressed as the means ± SEM for three separate experiments. DHE—Dihydroethidium; NADPH^p47^—NADPH oxidase subunit p47 (phox) and NQO1—NADPH:quinone oxidoreductase 1; GPx—Glutathione peroxidase. **P < .01, ***P < .005, ****P < .001

Both IgAN mice treated with saline (saline + IgAN) and Vehicle + IgAN mice had significantly higher levels of ROS in renal tissues, but this effect was significantly decreased in Tris DBA + IgAN mice (Figure 3A,B, Table 1). Similarly, although the percentage of the DHE‐positive cells was significantly increased in both saline + IgAN and Vehicle + IgAN mice compared with saline control or normal control mice treated with vehicle (vehicle control mice), this effect was markedly suppressed in Tris DBA + IgAN mice. In parallel, the levels of the NADPH oxidase p47phox (NADPH p47) and NQO1 were measured in renal tissues. As shown in Figure 3C, renal levels of NADPH p47 were significantly increased in both saline + IgAN and Vehicle + IgAN mice compared with those of saline control or vehicle control mice, but this effect was inhibited in Tris DBA + IgAN mice. Adequate activation of antioxidant pathway is a key mechanism in cellular defence against ROS production.^38^, ^39^ In contrast, greatly increased renal NQO1 was observed in Tris DBA + IgAN mice compared with that of saline + IgAN or Vehicle + IgAN mice (Figure 3C, Table 1). In consistence with these findings, Tris DBA + IgAN mice showed significantly elevated GPx compared with saline + IgAN or Vehicle + IgAN mice (Figure 3D, Table 1). Mitochondrial ROS plays an essential role in the process of NLRP3 inflammasome activation.^40^, ^41^ The cells were first treated Tris DBA for 1 hour and then co‐incubated with IgA‐ICs (Tris DBA + IgA‐ICs BMDMs). As shown in Figure 3E, mitochondrial ROS production was greatly reduced in Tris DBA + IgA‐ICs BMDMs compared with that of BMDMs treated with vehicle followed by IgA‐ICs.

### 3.4 Tris DBA reduced NLRP3 inflammasome activation in cultured macrophages

**Figure 4:**
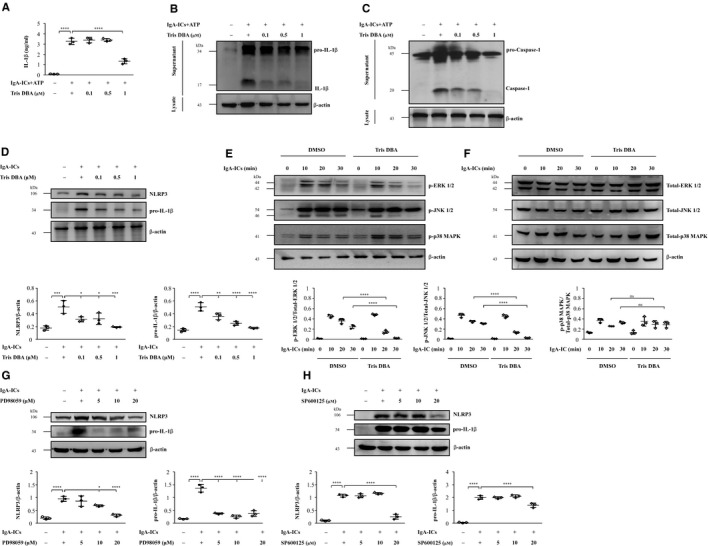
Tris DBA reduced the MAPK (ERK, JNK)‐mediated priming signal of the NLRP3 inflammasome in J774A.1 macrophages. A, IL‐1β secretion by J774A.1 macrophages measured by ELISA; B, IL‐1β and (C) caspase‐1 protein levels in J774A.1 macrophages assessed by Western blot analysis. J774A.1 macrophages incubated for 1 h with Tris DBA, then for 5.5 h with or without IgA‐ICs, and then for 30 min with or without 5 mmol/L ATP; D, NLRP3 and pro‐IL‐1β protein levels measured by Western blot and semiquantitative analysis. Cells incubated for 1 h with Tris DBA, then for 5.5 h with or without IgA‐ICs; E, JNK, ERK and p38 MAPK signalling pathways phosphorylation levels in J774A.1 macrophages measured by Western blot analysis and semiquantitative analysis; NLRP3 and pro‐IL‐1β protein levels measured by Western blot analysis. Cells incubated for 1 h with (F) PD98059 or (G) SP600125 then for 5.5 h with or without IgA‐ICs. PD98059, an ERK inhibitor, SP600125, a JNK inhibitor. The data are expressed as the means ± SEM for three separate experiments. ns, no difference, *P < .05, **P < .01, ***P < .005, ****P < .001

Recently, we found that full activation of the NLRP3 inflammasome requires both a priming signal such as IgA‐ICs and activation signal such as ATP to produce the mature IL‐1β.^10^ ATP, as an inducer for the activation signal of NLRP3 inflammasome activation, is required for IgA‐ICs‐mediated IL‐1β secretion.^10^ Next, the activation signal for NLRP3 inflammation activation was examined by determining the levels of caspase‐1 and IL‐1β using cultured macrophages. As shown in Figure 4, upon treatment with ATP (NLRP3 inflammasome activator), increased secretion levels of mature IL‐1β (Figure 4A,B) and caspase‐1 (Figure 4C) were observed in the Vehicle + IgA‐ICs macrophages compared to saline, but this effect was markedly inhibited in Tris DBA + IgA‐ICs macrophages.

#### 3.4.1 MAPK‐mediated priming signal of NLRP3 inflammasome

Cell viability was performed by MTT assay (Figure S1), which showed 1 µmol/L Tris DBA was the optimal dose for J774A.1 macrophages. The potential mechanism of action for Tris DBA to exert its inhibitory effect on NLRP3 inflammasome activation was evaluated in J774A.1. Recently, we showed that IgA‐ICs can enhance the expression of MAPK^9^ and activate NLRP3 inflammasome^4^, ^10^ in cultured macrophages. For their expression levels of NLRP3 and pro‐IL‐1β (priming signal), J774A.1 macrophages were treated with Tris DBA followed by IgA‐ICs before subjected to the test. As shown in Figure 4D, significantly reduced levels of the two proteins were seen in Tris DBA + IgA‐ICs macrophages compared to Vehicle + IgA‐ICs macrophages. However, there was no detectable proteins suggesting IL‐1β in the IgA‐ICs‐primed macrophages, indicating that the ICs without ATP could not further trigger the activation signal of NLRP3 inflammasome. MAPK has also been shown to be a potent activator for the priming signal of NLRP3 inflammasome.^42^ Although increased activation of MAPK signalling as demonstrated by the phosphorylation levels of ERK, JNK and p38 MAPK were observed in vehicle‐treated IgA‐ICs‐primed macrophages (Vehicle + IgA‐ICs macrophages), this effect was markedly inhibited in Tris DBA‐treated IgA‐ICs‐primed macrophages (Tris DBA + IgA‐ICs macrophages) (Figure 4E) and no significance difference in the expression levels of non‐phosphorylated ERK, JNK and p38 compared with vehicle‐treated IgA IC‐primed macrophages (Figure 4F). These findings suggest that Tris DBA treatment can negatively regulate the activation of NLRP3 inflammasome through inhibition of phosphorylated ERK and JNK but not non‐phosphorylated ERK and JNK. For further confirmation for the observation, two MAPK inhibitors, PD98059 (to ERK) and SP600125 (to JNK), were used, and treatment with them resulted in significantly reduced expression levels of NLRP3 and pro‐IL‐1β, compared to those without (Figure 4G,H). Also, we tested the activation of NF‐κB, a priming signal for NLRP3 inflammasome activation, in J774A.1 macrophages. Although increased NF‐κB activation was observed in Vehicle + IgA‐ICs compared to saline control, Tris DBA + IgA‐ICs failed to abolished this effect in the cells (data not shown).

#### 3.4.2 Autophagy‐mediated inhibition of NLRP3 inflammasome

**Figure 5:**
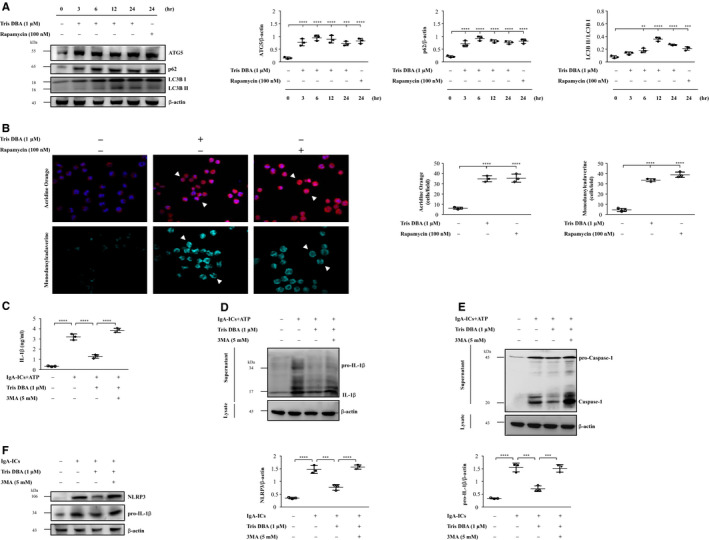
Tris DBA decreased the activation of NLRP3 inflammasome by autophagy. A, The expression levels of ATG5, p62 and LC3B I/II in the cells treated with Tris DBA (1 μmol/L) for 0, 3 ,6 12, 24 h and semiquantitative analysis of ATG5, p62 and LC3B I/II levels; B, AO and MDC staining; C, the effect of autophagy inhibitor, 3‐MA, on IL‐1β in IgA‐ICs and ATP primed macrophages and supernatant measured by ELISA and Western blot analysis of (D) IL‐1β and (E) caspase‐1. F, The effect of autophagy inhibitor, 3‐MA, on NLRP3 and pro‐IL‐1β in IgA‐ICs primed macrophages and analysis by Western blot. Original magnification, 400×. Arrow head indicates positive staining. AO—Acridine orange; MDC—monodansylcadaverine; Rapamycin was used as positive control for autophagy induction. Bars show the mean ± SEM for three separate experiments. **P < .01, ***P < .005, ****P < .001

Autophagy can inhibit priming and activation signals of NLRP3 inflammasome.^23^, ^24^, ^25^, ^26^ First, we examined whether Tris DBA could enhance autophagy induction in macrophages without IgA‐ICs stimulation. As shown in Figure 5A, treatment with Tris DBA significantly increased the autophagy protein markers of p62, ATG5 and LC3B I/II at 3 hours in J774A.1 macrophages and reached a plateau for the rest of time points. In consistence with the findings, J774A.1 macrophages treated with Tris DBA showed markedly enhanced formation of autophagic vacuoles as demonstrated by confocal microscopy with MDC or AO staining (Figure 5B). Next, we examined whether Tris DBA was able to inhibit the activation of NLRP3 inflammasome, including priming and activation signals, through prior induction of autophagy in J774A.1 macrophages. As shown in Figure 5C‐E, IL‐1β and caspase‐1 activation (activation signal) were observed in Tris DBA + IgA‐ICs macrophages compared to those of Vehicle + IgA‐ICs macrophages and significantly reduced expression levels of NLRP3 and pro‐IL‐1β production (priming signal), but this effect was restored by the 3‐MA, an autophagy inhibitor (Figure 5F).

Taken together, the results suggest that Tris DBA conferred its therapeutic effects on the IgAN mice by enhancing autophagy induction which in turn inhibited the priming and activation signals of NLRP3 inflammasome.

### 3.5 Tris DBA blunted the activating signal of NLRP3 inflammasome through SIRT1‐ and SIRT3‐mediated autophagy induction in cultured macrophages

**Figure 6:**
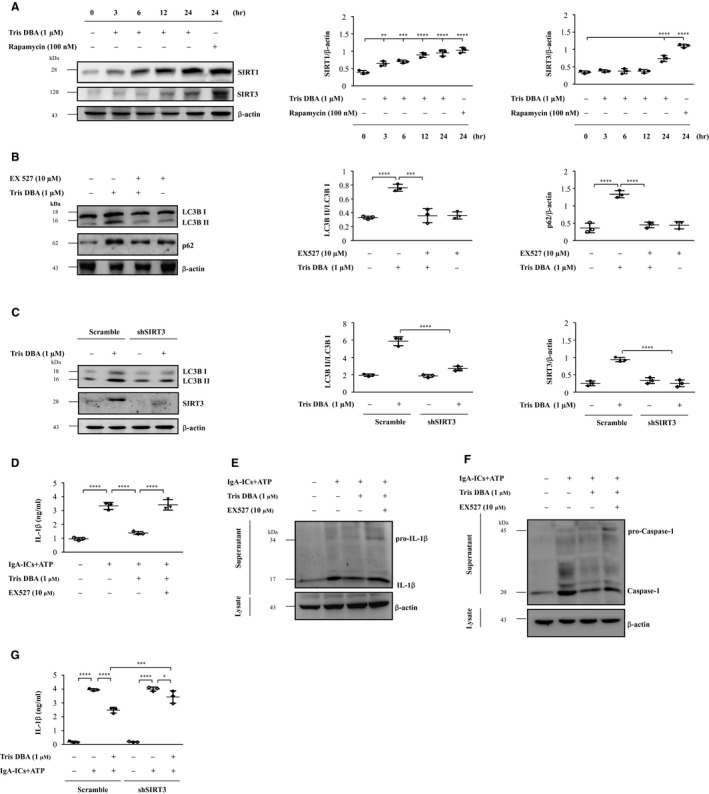
Tris DBA inhibits the activating signal of NLRP3 inflammasome through SIRT1‐ and SIRT3‐mediated autophagy induction in J774A.1 macrophages. A, Western blot analysis for SIRT1 and SIRT3 levels in the cells treated with Tris DBA (1 μmol/L) for 0, 3 ,6 12, 24 hours and semiquantitative analysis of SIRT1 and SIRT3 levels; B, the effect of SIRT1 inhibitor, EX527, on p62 and LC3B I/II in Tris DBA primed macrophages; C, The effect of SIRT3 shRNA on LC3B I/II and SIRT3 in Tris DBA primed macrophages; D, The effect of EX527 on IL‐1β secretion in IgA‐ICs + ATP primed macrophages measured by ELISA and (E) IL‐1β and (F) caspase‐1 protein levels in J774A.1 macrophages assessed by Western blot analysis. G, The effect of shSIRT3 on IL‐1β secretion in IgA‐ICs + ATP primed macrophages measured by ELISA. Bars show the mean ± SEM for three separate experiments. *P < .05, **P < .01, ***P < .005, ****P < .001

SIRT1 and SIRT3 have been reported to enhance the autophagy.^27^ As shown in Figure 6A, treatment with Tris DBA significantly increased the protein expression of SIRT1 starting from 6 hours and persisted until 24 hours, and SIRT3 at 24 hours in J774A.1 macrophages in a dose‐dependent manner. Next, the cells were first treated with EX‐527, an inhibitor of SIRT1, followed by the treatment with Tris DBA or shSIRT3 macrophages to elucidate whether SIRT1 or SIRT3 was involved in autophagy induction in the macrophages treated with Tris DBA. As shown in Figure 6B,C, Tris DBA induced significantly increased expression of LC3B I/II and p62 in the cells. Moreover, this effect was abolished in the macrophages deficient in SIRT1 and SIRT3. In addition, although reduction of IL‐1β secretion and caspase‐1 was observed in Tris DBA + IgA‐ICs macrophages, this effect was reversed by the administration of EX‐527 and shSIRT3 (Figure 6D‐G), respectively.

## 4 DISCUSSION

In the present study, we show the therapeutic effect of Tris DBA on IgAN in mice, the mechanism of action involving inhibition of MAPK signalling pathways and blunting of ROS‐mediated inflammatory reactions and enhancing SIRT1‐ and SIRT3‐mediated autophagy induction and autophagy‐mediated NLRP3 inflammasome suppression. In the past, we demonstrated that overproduction of ROS activates NLRP3 inflammasome as a key mechanism underlying the development of the mouse IgAN model. This includes the following: (a) inflammasome activation is mediated by ROS by using ROS inhibitors^10^, ^29^; (b) decreased ROS production and increased activation of the NrF2 antioxidant pathway attenuates renal pathology^4^, ^8^; and (c) inhibited NLRP3 inflammasome activation and levels of ROS, NAD(P)H oxidase subunit p47phox, or COX‐2, and activated NrF2 is beneficial to an autoimmune renal condition in the mice. Next, SIRT1 and SIRT3 have been shown to inhibit the mitochondrial ROS production in skin,^43^ myocardium^44^, ^45^ or the kidney^46^, ^47^ and both play a crucial role in autophagy induction.^27^ Therefore, we further examined the effect of Tris DBA on SIRT1 and SIRT3 expression (Figure 6A) showing increased production of these proteins was compatible with the induction of autophagy (Figure 6B,C), and these findings were clearly associated with the reduction of NLRP3 and IL‐1β in renal tissues (Figure 2) or macrophages after Tris DBA treatment when compared to the vehicle treatment (Figure 6D‐G), which may be part of the mechanism responsible for the effect of Tris DBA in IgAN.

To evaluate the role of Tris DBA on signalling downstream of IC deposition, we examined the effect of this compound on macrophages treated with IgA‐ICs. We demonstrated that Tris DBA treatment can induce autophagy in macrophages at 3 to 24 hours (Figure 5). This finding was confirmed by reduced expression of the priming and activation signals of the NLRP3 inflammasome in IgA‐ICs‐primed macrophages treated with Tris DBA, but the activation of the inflammasome was restored by treatment with 3‐MA, an autophagy inhibitor (Figure 5C‐F). All the findings obtained in vitro clearly indicated a beneficial effect of using Tris DBA to treat IgAN mice, in view that greatly improved renal condition was observed in the treated mice. Consistent with the in vitro experiments, we clearly revealed dramatically increased autophagy expression as early as the first day after the administration of the compound, which persisted for at least three days, in saline mice treated with Tris DBA as a comparative experiment to confirm the augmented induction of autophagy and resultant potential therapeutic effect on the IgAN mice that were treated with Tris DBA in the study (Figure 2).

In our previous study, we demonstrate that ATP contributed the NLRP3 inflammasome‐mediated IL‐1β production after treated with IgA‐ICs in antigen presenting cells by dose‐dependent manner.^10^ In this regard, we found that ATP, as an inducer for the activation signal for NLRP3 inflammasome activation, is required for IgA‐ICs‐mediated IL‐1β secretion in macrophages. In addition, it should be noted that IgA1 deposition may induce NLRP3 expression and macrophage transdifferentiation of podocyte in IgAN.^11^ Thus, the activation of NLRP3 inflammasome might contribute by both ATP and IgA1 and trigger the renal inflammation through renal cells and macrophages in IgAN. In addition, infiltration of macrophages into the kidney is a hallmark of IgAN,^7^, ^9^, ^10^ in which these mononuclear leucocytes initiate the inflammatory response in renal tissues and resultant injury in the disease. In our previous study, we show that IgA‐ICs triggered ROS production and activation and may involve in NLRP3 inflammasome activation to contribute the development and progression of IgAN. However, whether other types of renal intrinsic cells such as podocytes, mesangial cells and any other kidney cells are worth further investigation for their roles involved in the mechanism of action for the therapeutic effects of Tris DBA. On the other hand, NLRP3 inflammasome activating signals such as mtDNA, ROS and cytosolic presentation of cardiolipin, all of which can serve as direct activators of the inflammasome. Recently, we demonstrated that IgA‐ICs enhances the MAPKs signalling activation (as priming signal) and mtROS production (as activation signal) to stimulate the NLRP3 inflammasome‐mediated IL‐1β secretion in macrophages primed with the ICs.^9^, ^10^ Thus, the fact that Tris DBA enhanced the autophagy induction could inhibit not only the MAPKs signalling activation but also decrease the mtROS production, thereby inhibiting the NLRP3 inflammasome activation.

However, a reduced induction of autophagy was observed in renal tissues of Tris DBA + IgAN mice (Figure S2) in contrast to the observation of increased autophagy induction in renal tissues obtained from normal mice treated with Tris DBA (Figure 2). In this regard, autophagy might represent a protective mechanism or a feedback regulation under the conditions of the renal disease. In other words, it is likely that Tris DBA treatment exerted autophagy induction activity under normal conditions, which helped to prevent disease development or progression. However, Tris DBA treatment ameliorated the effect in IgAN mice, presumably followed by a decreased autophagic response due to the improved renal conditions caused by treatment with the compound. Although the exact mechanism is worth further investigation under the disease condition, the emergence of complicated interactions among autophagy induction remains uncertain, and NLRP3 inflammasome activation and compensatory reactions might have occurred in IgAN mice. Our hypothesis can be supported by the following findings.^1^ In response to Tris DBA treatment, while in macrophages treated with both Tris DBA and IgA IC, the use of an inhibitor of autophagy (3‐MA) restored the levels of caspase‐1 activation and IL‐1β secretion (Figure 5), suggesting that the treatment with Tris DBA could induce autophagy in the cells treated with IgA IC.^2^ Increased autophagy induction was observed in renal tissues obtained from untreated normal mice with Tris DBA (Figure 2).^3^ Decreased autophagy induction could also represent a negative feedback during the improvement of the mouse model of IgAN for the study period. In addition, the expression of p62, another autophagy marker, is regulated by the balance between transcriptional regulation (incoming flux) and post‐translational autophagic degradation (outgoing flux) of the protein.^48^ In the current study, Tris DBA may exert its effect at the level of both the synthesis and degradation of autophagy in macrophages.

It is established that ATG5 plays a crucial role in the membrane elongation and dissociate from the membrane upon completion of autophagosome formation.^49^ The isoform LC3B I is synthesized in the cytosol, and after conjugation to phosphatidylethanolamine, the activated isoform LC3B II is located within the autophagosomal membrane, followed by its disruption after autolysosome formation.^50^ Consistent with this notion, in the present study, increased expression of LC3B II was observed at days 1, 3 and 7, but not at day 28 in the mice treated with Tris DBA (Figure 2C), suggesting that LC3B II was produced more than disrupted at early stage, while the production of LC3B II was reduced due to degradation through lysosomal hydroplanes at day 28. Besides, the ATG5 expression was opposite to the LC3B II in the mice. Therefore, the underlying mechanism deserves further investigation.

**Figure 7:**
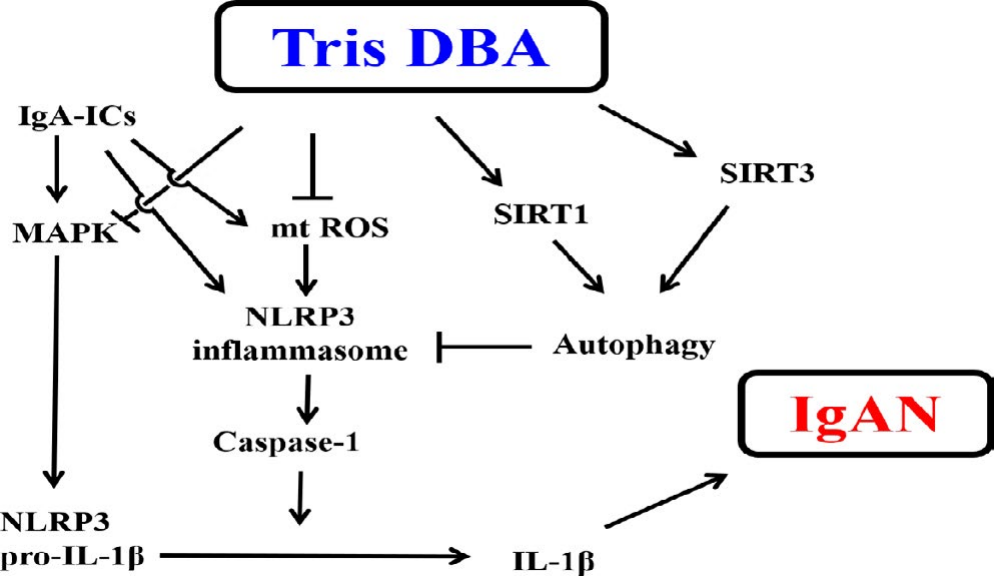
Schematic representation for the plausible mechanism of action of therapeutic effects rendered by Tris DBA. IgA‐ICs, IgA immune complexes; ROS, reactive oxygen species; SIRT1, sirtuin 1; SIRT3, sirtuin 3, mtROS, mitochondrial ROS

As illustrated in Figure 7, it is suggested that Tris DBA was able to effectively ameliorate the mouse IgAN model. This beneficial effect might involve blunting of mitochondrial ROS production, a MAPK (ERK, JNK)‐mediated priming signal of the NLRP3 inflammasome and differentially regulating the autophagy/NLRP3 inflammasome axis through SIRT1 and SIRT3. Thus, further development of Tris DBA as a therapeutic candidate for IgAN is warranted.

## CONFLICT OF INTEREST

All the authors have no competing interests.

## AUTHOR CONTRIBUTIONS

**Chung‐Yao Wu:** Conceptualization (lead); data curation (lead); formal analysis (lead); investigation (lead); methodology (lead); project administration (lead); writing‐original draft (lead). **Kuo‐Feng Hua:** Formal analysis (equal); methodology (lead); project administration (supporting); writing‐review and editing (equal). **Shin‐Ruen Yang:** Data curation (equal); investigation (equal); project administration (equal). **Yi‐Shan Tsai:** Data curation (equal); methodology (equal); project administration (equal). **Shun‐Min Yang:** Conceptualization (supporting); methodology (supporting); writing‐review and editing (supporting). **Chih‐Yu Hsieh:** Data curation (equal); methodology (equal); project administration (equal). **Chia‐Chao Wu:** Methodology (equal); visualization (equal); writing‐review and editing (equal). **Jia‐Feng Chang:** Methodology (equal); project administration (equal); supervision (equal). **Jack L. Arbiser:** Supervision (equal); writing‐review and editing (equal). **Chiz‐Tzung Chang:** Writing‐review and editing (equal). **Ann Chen:** Data curation (lead); investigation (lead); project administration (equal); resources (lead); supervision (equal); writing‐original draft (equal); writing‐review and editing (lead). **Shuk‐Man Ka:** Methodology (lead); project administration (lead); resources (supporting); supervision (equal); writing‐review and editing (lead).

## Supporting information

Fig S1‐S2

## Data Availability

The data that support the findings of this study are available on request from the corresponding author. The data are not publicly available due to privacy or ethical restrictions.
