# Correction to ‘Tris DBA Ameliorates IgA Nephropathy by Blunting the Activating Signal of NLRP3 Inflammasome Through SIRT1‐and SIRT3‐Mediated Autophagy Induction’

**DOI:** 10.1111/jcmm.70502

**Published:** 2025-05-01

C. Y. Wu, K. F. Hua, S. R. Yang, et al., “Tris DBA Ameliorates IgA Nephropathy by Blunting the Activating Signal of NLRP3 Inflammasome Through SIRT1‐ and SIRT3‐Mediated Autophagy Induction,” *Journal of Cellular and Molecular Medicine* 24 (2020): 13609–13622, 10.1111/jcmm.15663.

In Chung‐Yao Wu et al. the DHE immunofluorescence staining for Saline + IgAN mice in Figure 3B was mistakenly omitted during image preparation. The correct figure is shown below. The authors confirm all results and conclusions of this article remain unchanged.

**Correct image (Saline ± IgAN in Figure 3B):**

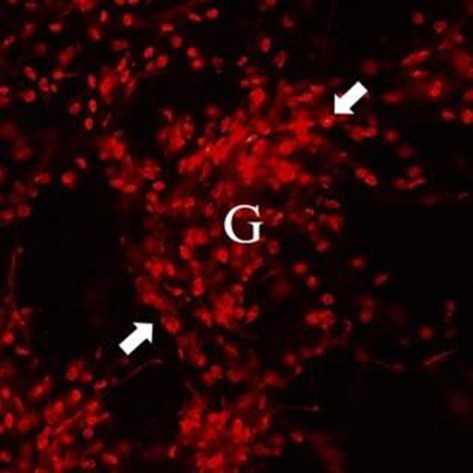

**Revised image (Figure 3):**

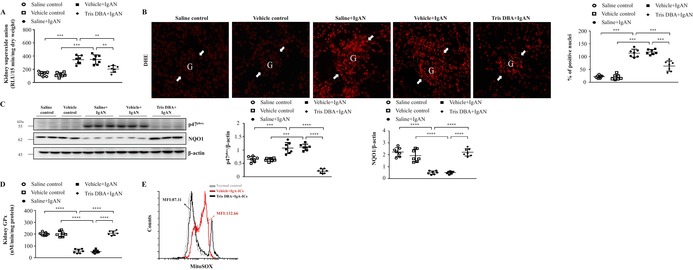

**FIGURE 3** Tris DBA reduced oxidative stress and enhanced ROS activity in IgAN mice. (A) Superoxide anion in renal tissues; (B) DHE staining on renal tissues and the quantified results of DHE staining. Original magnification, 400×. Arrow indicates glomerular area; (C) the protein level of NADPH^p47^ and NQO1 in Western blot analysis in renal tissue and semiquantitative analysis; (D) renal GPx level in ELISA. Bars show the mean ± SEM results in seven mice per group. (E) Mitochondrial ROS levels in BMDMs measured by MitoSOX after Tris DBA treatment, priming with IgA‐ICs for 5.5 h and stimulation with ATP. The data are expressed as the means ± SEM for three separate experiments. DHE, Dihydroethidium; GPx, Glutathione peroxidase; NADPH^p47^, NADPH oxidase subunit p47 (phox); NQO1, NADPH:quinone oxidoreductase 1. **p < 0.01, ***p < 0.005, ****p < 0.001.

