# Supplementary material for: Tris DBA ameliorates IgA nephropathy by blunting the activating signal of NLRP3 inflammasome through SIRT1‐ and SIRT3‐mediated autophagy induction
Source: J Cell Mol Med. 2020 Nov 1;24(23):13609–22. doi: 10.1111/jcmm.15663 (PMC7753881; doi:10.1111/jcmm.15663)
Supplement: Supplementary file 1 — Fig S1‐S2 [file JCMM-24-13609-s001.pdf]

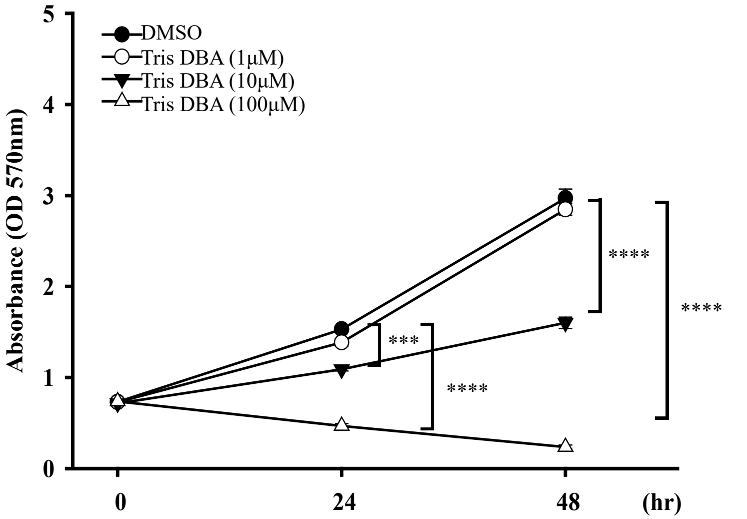

Figure S1. Cell viability of Tris DBA in J774A.1 macrophages by MTT assay. Data show the mean ± SEM for three separate experiments. ***p<0.005, ****p<0.001.

Figure S1. Wu *et al.*

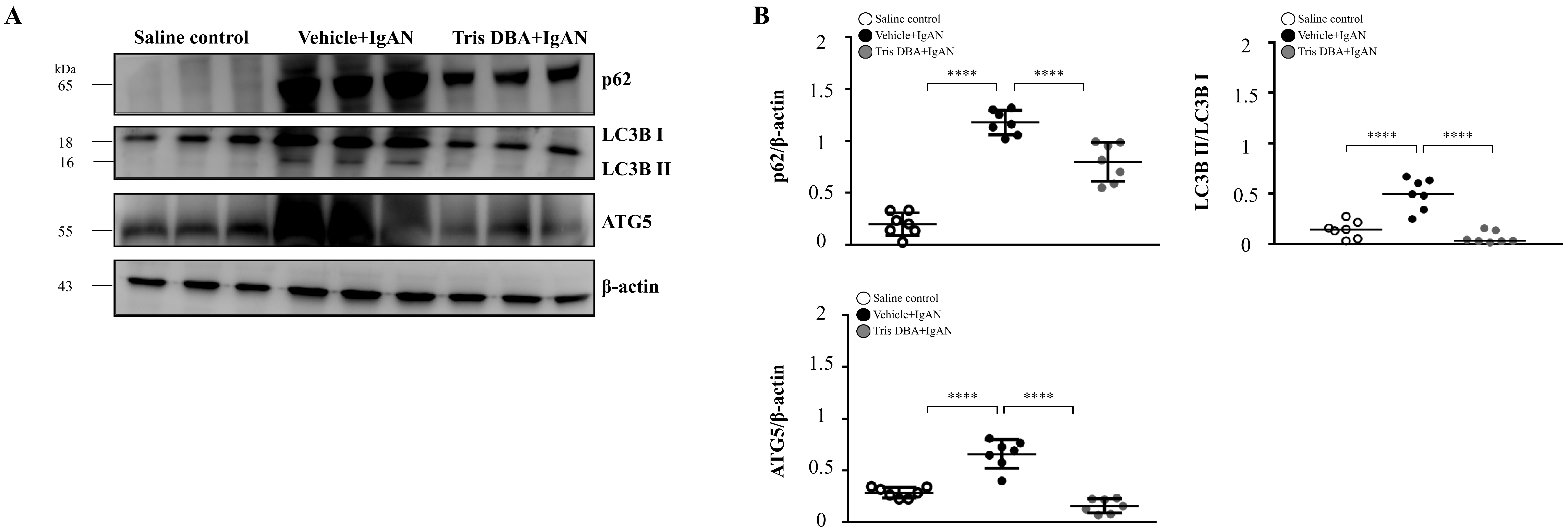

Figure S2. Renal autophagy induction in IgAN. The expression levels of (A) p62, LC3B, ATG5 in Western blot analysis and (B) Semiquantitative analysis. Data show the mean ± SEM results in 7 mice per group. ****$p<0.001$.

Figure S2. Wu *et al.*
